# Optimized removal of hexavalent chromium from water using spent tea leaves treated with ascorbic acid

**DOI:** 10.1038/s41598-022-12787-0

**Published:** 2022-05-25

**Authors:** Qammer Zaib, Daeseung Kyung

**Affiliations:** School of Civil and Environmental Engineering, University of Ulsan, Daehak-ro 93, Nam-gu, Ulsan, 44610 Republic of Korea

**Keywords:** Pollution remediation, Environmental sciences, Engineering

## Abstract

Spent tea leaves were functionalized with ascorbic acid to obtain treated tea waste (t-TW) to encourage the adsorption of hexavalent chromium from water. The adsorption removal of Cr(VI) was systematically investigated as a function of four experimental factors: pH (2–12), initial Cr(VI) concentration (1–100 mg L^−1^), t-TW dosage (0–4 g L^−1^), and temperature (10–50 °C) by following a statistical experimental design. A central composite rotatable experimental design based on a response surface methodology was used to establish an empirical model that assessed the individual and combined effects of factors on adsorptive removal of Cr(VI). The model was experimentally verified and statistically validated then used to predict optimal adsorption removal of Cr(VI) from water. At optimized conditions, ≥ 99% of 1 mg L^−1^ Cr(VI) can be removed by 4 g L^−1^ t-TW at a pH of 9. The adsorptive mechanism was assessed by conducting kinetics and equilibrium studies. The adsorption of Cr(VI) by t-TW followed a pseudo-second-order kinetics model (k_2_ = 0.001 g mg^−1^ h^−1^) and could be described by Langmuir and Temkin isotherms, indicating monolayer adsorption and predominantly adsorbate-adsorbent interactions. The t-TW exhibited a competitive Cr(VI) adsorption capacity of 232.2 mg g^−1^ compared with the other low-cost adsorbents. These results support the utilization of tea waste for the removal of hazardous metal contaminants from aqueous systems.

## Introduction

Chromium is among the more widespread of heavy metals in aquatic ecosystems, in part due to wastewater released from the chromium plating, leather tannery, textile, and wood preservation industries^1^. In environmental systems, chromium is present primarily in hexavalent and trivalent oxidation states. Cr(III) is generally non-toxic and is an essential agent in animal and human metabolisms^2^. However, Cr(VI) is hazardous to living organisms due to its mutagenicity and carcinogenicity^2^ and is among 129 critical pollutants identified by the US EPA^3^. Cr(VI) is known to induce lung cancer, skin irritation, and damage to kidneys, livers, and gastric systems^2,4^. Because wastewater is one of the major environmental sources of this pollutant, the EPA and WHO have limited the maximum permissible levels of chromium to 0.1 and 0.05 mg L^−1^, respectively^5,6^. It is therefore highly desirable to bring high concentrations of Cr(VI) in wastewater down to allowable limits before releasing it to the environment.

Concentrations of Cr(VI) and other heavy metals in water can be controlled by ion exchange, solvent extraction, chemical precipitation, membrane-based separation, and adsorption^7–10^. Among these technologies, adsorption is popular due to its efficiency, convenience, smaller land footprint, and simplicity of operation^8,11^. However, its commercial success is limited by high costs associated with the need for periodic replacement of adsorbent material. The cost-effectiveness of adsorption can be improved by utilizing low-cost adsorbent materials, including waste and biomass^8,12^. Tea waste (TW) in particular has demonstrated an ability to effectively adsorb Cr(VI) from aqueous systems^13,14^.

Tea waste is an intriguing adsorbent due to the prevalence of functional groups on its surface^13,15^. These functional groups can be fine-tuned using chemical treatments according to the intended use, including the adsorptive removal of Cr(VI). Tea is one of the most consumed beverages worldwide. With a 4.9% annual growth rate, tea production is estimated to reach 8.52 million tonnes by the year 2025^16^. Black tea in particular constitutes approximately 87% of all the tea consumed in the US and between 75 and 80% of worldwide consumption^16^, leading to the production of large volumes of TW. This issue can be mitigated by utilizing black TW as an adsorbent material. The utilization of TW as an adsorbent not only contributes to circular economies and low environmental impacts, but may help achieve United Nation Sustainable Development Goal 12.5: “By 2030, substantially reduce waste generation through prevention, reduction, recycling and reuse”^17^.

In recent years, researchers have investigated the potential of TW as an adsorbent for Cr(VI) removal^13,18^. These studies have varied one factor at a time while keeping the others constant, an approach that helps reveal the adsorption mechanisms (e.g., equilibrium, kinetics, and thermodynamics) but provides little understanding of the combined impact of these factors in real-world applications. This problem can be attenuated by systematically investigating the combined effects of factors through statistical experimental design and a response surface methodology (RSM), which is a combination of statistical and mathematical techniques. To the best of our knowledge, no studies have been reported in which black TW functionalized with ascorbic acid was optimized for the removal of Cr(VI) from water at varying conditions of pH, initial Cr(VI) concentrations, adsorbent dosage, and temperature.

In this study, black TW was functionalized with ascorbic acid to synthesize treated tea waste (t-TW). Ascorbic acid is a green reducing agent capable of reducing oxygenated functional groups to help t-TW adsorb Cr(VI). It was selected as a functionalizing agent due to its lack of toxicity, low cost, and ability to protect living organisms from genotoxic effects induced by hazardous metal ions^19^. Ascorbic acid largely reduces hydroxyl (–OH), epoxy (C–O–C), carboxyl (–COOH), and carbonyl (C=O) functional groups on adsorbent materials such as those used in our study^20^. The t-TW was characterized and tested for adsorption removal of Cr(VI) from water. An RSM-based central composite rotatable experimental design (CCRD) was used to model Cr(VI) adsorptive removal from water at variable pH values, initial Cr(VI) concentrations, adsorbent dosages, and temperatures. The effect of the variables and their interactions on the adsorption process was studied and optimal parameters for the adsorptive removal of Cr(VI) were identified. The adsorption kinetics and equilibrium isotherms were also studied to better understand the adsorption mechanism of Cr(VI) in aqueous phase.

## Materials and methods

### Materials

Potassium dichromate (≥ 99.5%), l-ascorbic acid (≥ 99.0%), sodium hydroxide (≥ 98.0%), and hydrochloric acid (37.0%) were purchased from Sigma-Aldrich. Sulfuric acid (> 95.0%) and 1,5-diphenylcarbohydrazide (DPC) (≥ 98.0%) were obtained from Kanto Chemical Co., Inc. (Japan). Acetone (≥ 99.5%) was supplied by OIC Co., Ltd. (Korea). A 100 mL solution of the DPC reagent was prepared by dissolving 500 mg of DPC in 100 mL of acetone. Distilled deionized water (DIW) with an average resistivity of 18.2 MΩ-cm, was used to prepare stock and working solutions.

### Synthesis of treated tea waste

Tea waste was obtained from spent black tea bags produced by Lipton Yellow Label (Unilever Russia, St Petersburg, Progonnaya, UL, 1). In a typical experiment, once-used black tea bags (20 g, 10 bags of 2 g each) were emptied into a glass beaker. Boiling DIW (500 mL at 100 °C) was poured into the beaker to soak the TW. After 10 min, the water was decanted and replaced with freshly boiled water. This procedure was repeated six times to ensure the removal of all water-soluble compounds (including color) from the TW. The washed TW was dried (60 °C) in a drying oven overnight (≥ 12 h) and then stored in a desiccator until further use. The functionalizing agent was prepared by dissolving ascorbic acid (4.4 g) in DIW to obtain a 0.5 M aqueous ascorbic acid solution (50 mL), which was added to 10 g of TW and mixed thoroughly. The mixture was covered and heated for 8 h in a laboratory oven. The obtained t-TW was washed three times with DIW to dissolve any residual ascorbic acid. The final product (t-TW) was dried in a drying oven (60 °C) until a constant weight was reached (≥ 12 h). The adsorbents were stored in airtight containers until used.

### Adsorbent characterization

A JSM-6500 scanning electron microscope (SEM; JEOL, Japan) and energy-dispersive X-ray spectroscopy (EDX) was used to characterize the morphological structure and elemental composition of the adsorbents. The microscope was operated at 3–15 kV to obtain the micrographs and EDX spectrum. Fourier transform infrared (FTIR) spectroscopy (Nicolet iS5, Thermo Fisher Scientific, US) was performed using an attenuated total reflection detector to assess functional groups present on the material in a wavelength range of 400–4000 cm^−1^ using KBr pellet samples.

### Experimental design and model development

A CCRD based on an RSM was selected to study the individual, synergistic, and antagonistic effects of four factors (variables) on response, i.e., Cr(VI) adsorption removal (%) from water. Design Expert software was used for statistical analysis. The four studied factors were: pH of the aqueous system (A), initial Cr(VI) concentration in mg L^−1^ (B), t-TW (adsorbent) dosage in g L^−1^ (C), and temperature in °C (D). The experimental factors and their levels are presented in Table 1. The central, factorial, and axial points are represented by 0, ± 1, and ± α, respectively, where α represents the distance between the center and axial points. Its value is equal to (2^k^)^¼^ where k represents the number of experimental factors.

**Table 1:** Experimental factors for the adsorptive removal of Cr(VI) from water using tea waste treated with ascorbic acid.

	Factors	Units	Levels				
			− α	− 1	0	1	+ α
A	pH	–	2.0	4.5	7.0	9.5	12.0
B	Cr(VI) conc.	Mg L^−1^	1.0	25.8	50.5	75.3	100.0
C	Treated tea waste (t-TW)	G L^−1^	0.0	1.0	2.0	3.0	4.0
D	Temperature	°C	10.0	20.0	30.0	40.0	50.0

A complete experimental design matrix (Table 2) shows a combination of factors—required by CCRD to construct an empirical model—to investigate the impact of each factor on Cr(VI) adsorption. For the four variables in the experiments, 30 experimental combinations were evaluated (Table 2)—16 at factorial points, 8 at axial points and 6 (replicates) at the central points. Six replicates at the central point were chosen (instead of three) to best estimate experimental error, ensure the robustness of the model, and capture maximum variability^21^. Details for the procedure of the adsorption experiments are presented in following section.

**Table 2:** Experimental design matrix and response of experimental settings for the adsorptive removal of Cr(VI) by tea waste treated with ascorbic acid.

Run	Factors				Response	
	A	B	C	D	Cr(VI) adsorption removal (%)	
	pH	Cr(VI) (mg L^−1^)	t-TW (g L^−1^)	Temp. (°C)	Experimental	Predicted
1	7.0	50.5	0.0	30.0	0.0	0.4
2	9.5	75.3	1.0	40.0	10.1	13.9
3	9.5	25.8	3.0	20.0	19.3	22.8
4	4.5	75.3	1.0	40.0	25.4	25.4
5	9.5	25.8	1.0	20.0	1.3	2.5
6	4.5	25.8	1.0	40.0	26.0	31.8
7	9.5	75.3	1.0	20.0	6.9	7.9
8	9.5	25.8	3.0	40.0	27.5	28.8
9	7.0	50.5	2.0	10.0	10.7	5.9
10	9.5	25.8	1.0	40.0	9.1	8.5
11	7.0	1.0	2.0	30.0	66.0	63.2
12	4.5	25.8	3.0	20.0	47.8	50.3
13	7.0	50.5	2.0	30.0	9.5	9.6
14	7.0	50.5	2.0	30.0	10.2	9.6
15	7.0	50.5	2.0	30.0	12.1	9.6
16	9.5	75.3	3.0	20.0	14.6	14.3
17	7.0	100.0	2.0	30.0	5.9	3.1
18	4.5	25.8	1.0	20.0	17.7	16.8
19	7.0	50.5	2.0	30.0	8.9	9.6
20	12	50.5	2.0	30.0	4.7	2.0
21	4.5	75.3	3.0	40.0	43.6	45.0
22	2.0	50.5	2.0	30.0	93.3	90.5
23	7.0	50.5	4.0	30.0	18.3	18.7
24	9.5	75.3	3.0	40.0	19.3	20.3
25	4.5	75.3	3.0	20.0	26.6	30.0
26	7.0	50.5	2.0	30.0	11.1	9.6
27	4.5	25.8	3.0	40.0	67.1	65.3
28	7.0	50.5	2.0	50.0	18.2	13.3
29	7.0	50.5	2.0	30.0	7.7	9.6
30	4.5	75.3	1.0	20.0	9.7	10.4

### Adsorption experiments

Five sets of adsorption experiments were performed: (1) preliminary experiments to observe the adsorption difference between TW and t-TW and screen the important factors and ranges effecting the adsorption removal of Cr(VI) by t-TW; (2) thirty experiments according the RSM experimental design (Table 2) to construct the RSM model, (3) three experiments to validate the predictability of RSM model at optimized conditions (≥ 99% removal of Cr(VI) from water); (4) six experiments to study the equilibrium adsorption of Cr(VI) on t-TW; and (5) twelve experiments to study the adsorption kinetics. All experiments were performed in duplicate (at least) and average values were reported.

A stock solution of Cr(VI) (1000 mg L^−1^) was prepared by dissolving 2.83 g of potassium dichromate in 1 L of DIW. The working solutions were prepared from the stock solution by subsequent dilutions. The adsorption experiments were performed by adding pre-determined amounts of adsorbent in a 40 mL amber vial followed by the addition of the Cr(VI) solution. The vials were shaken at 200 rpm using an incubator shaker (Intron Biotechnology, Korea). The residual concentrations of Cr(VI) were determined by following the 1,5 DCP method^22^ using an Ultraviolet–Visible light spectrophotometer (Genesys 10S Vis by Thermo Scientific) and the following equation:

1 $$ {\text{Removal }}\left( \% \right) \, = \, \left( {{\text{C}}_{{\text{i}}} - {\text{C}}_{{\text{f}}} } \right) \, /{\text{ C}}_{{\text{i}}} \times {1}00, $$

where C_i_ and C_f_ are the initial and final concentrations of Cr(VI), respectively.

The experimentally obtained equilibrium data were fitted to adsorption isotherms such as Langmuir, Freundlich, Temkin, and Dubinin–Radushkevich (D–R) models. The kinetics data were evaluated through pseudo-first-order (PFO), pseudo-second-order (PSO), and intraparticle diffusion models. Nonlinear regression analyses were applied for adsorption isotherm models^23^ and adsorption kinetics models^24^. Linear regression, while convenient, introduces error propagation and inaccurately estimates model parameters^23^. The model parameters were therefore calculated by nonlinear solving methods using Microsoft Excel Solver. The fittings of the regression models were evaluated to gauge the model fitting accuracy.

## Results and discussion

### Characterization

**Figure 1:**
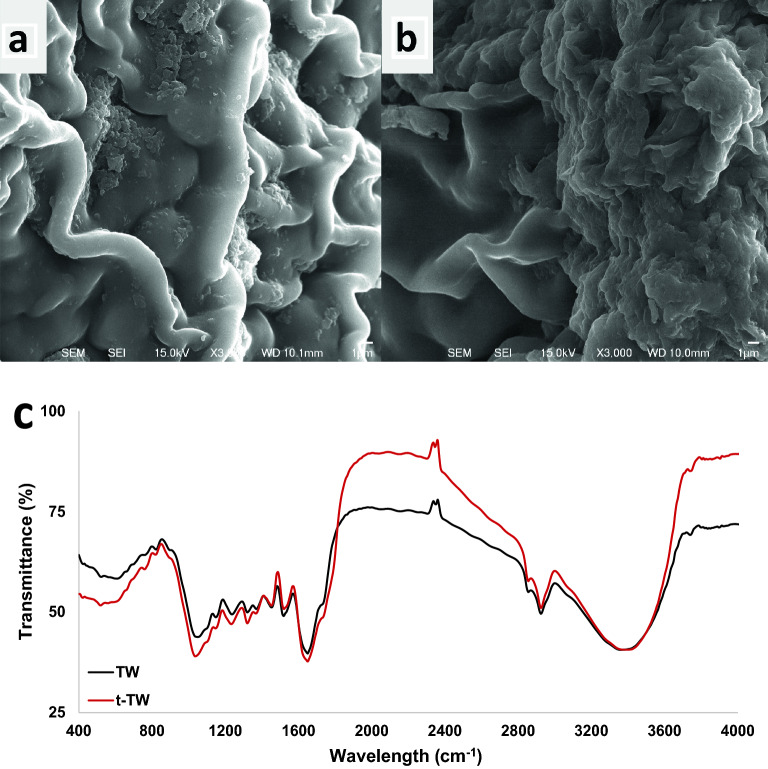
SEM of (**a**) tea waste (TW) and (**b**) treated tea waste (t-TW) modified with ascorbic acid. (**c**) FTIR spectra of the adsorbents.

The morphology of TW and t-TW was characterized using an SEM, which generates a beam of electrons that interact with the surface of a sample to provide information about the topography and composition of a sample^25,26^. Figure 1a,b depicts SEM images of the surface characteristics of TW and t-TW, respectively. Both images represent a largely smooth surface at the same (× 3000) magnification. There were no observable differences before and after treatment with ascorbic acid, other than a slight increase in surface roughness in the t-TW. However, EDX analysis revealed a slight decrease in oxygen content from 27.7 in TW to 25.5% in t-TW. This may be due to reduction of some oxygen-rich functional groups^27^. As previous studies have suggested the biosorption of heavy metals by carboxyl and amine groups present on similar materials^13,28^, FTIR analysis was performed, as shown in Fig. 1c.

**Figure 2:**
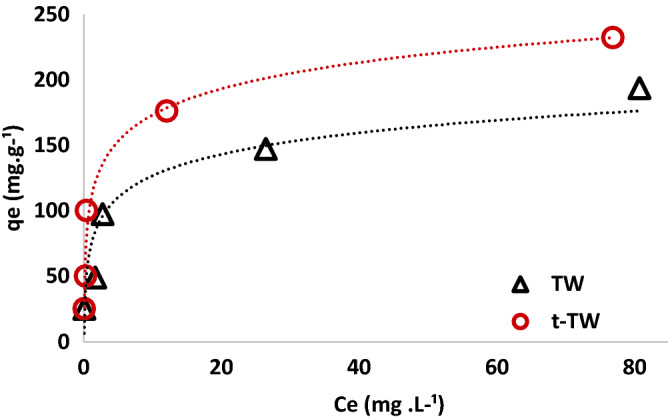
Adsorption of Cr(VI) on tea waste (TW) and ascorbic acid treated tea waste (t-TW). (Contact time: 48 h, initial pH: 6.5, initial Cr(VI) concentration: 100 mg L^−1^; adsorbent dosage: 0.1–4 g L^−1^, and temperature: 25 °C).

The TW and t-TW comprise numerous functional groups that can adsorb Cr(VI)^13,25,28–30^. It has been reported that the absorbance at certain wavelengths varied by magnitude and/or shifted following ascorbic acid treatment of TW to synthesize t-TW; the absorbance increased near wavelengths of 1040, 1060, and 2873 cm^−1^ but decreased at 1500, 1800–3200, and > 3550 cm^−1^. In Fig. 1c, the absorbance bands between 600 and 900 cm^−1^ represent primary and secondary amines and amides; those near 1040 cm^−1^ represent polysaccharides and aromatic ethers (C–O–C) and silicates (Si–O–Si); those at 1319 cm^−1^ represent C–N stretching of primary and secondary aromatic amines; those at 1420 cm^−1^ represent C–O stretching of carbonates and/or O–H plane bending of carboxylic acid; those at 1650 cm^−1^ represent C=C stretching of carboxylic acid and amide I; those between 1600 and 1750 cm^−1^ represent C=O vibration of bonded conjugated aldehydes, ketones, esters, and ketones; those between 2850 and 2920 cm^−1^ represent C–H stretching of aliphatic groups; and those near 3400 cm^−1^ represent either O–H stretching of hydroxyl groups (i.e. carboxylic acids, alcohols, and phenols) or N–H stretching in primary amines, secondary amines, and/or amides^25,29,30^. The antioxidant activity of black tea was enhanced by ascorbic acid^31^. This can be attributed to the lessening of carbonyl, epoxy, aromatic ethers, carboxyl, and hydroxyl functional groups due to partial reduction of t-TW by ascorbic acid as reported previously^20,32^. These variabilities in functional groups lead to the comparatively enhanced adsorption of Cr(VI) from water by t-TW, as shown in Fig. 2. The experimentally observed equilibrium adsorption capacity of t-TW was up to 20 percent greater than that of TW at identical shaking speeds, temperatures, pH values and water quality. The observed maximum experimental adsorption capacities of TW and t-TW were 193.45 mg g^−1^ and 231.95 mg g^−1^, respectively. These values are comparable to those reported for mixed tea waste^13^ and tea-waste biochar^14^. These results warranted further investigation into modeling and optimization of Cr(VI) adsorption by t-TW.

### Model development, validation, and diagnostic analysis

The measured Cr(VI) removal (%) at different pH, initial Cr(VI) concentration, t-TW dosage, and temperature settings are listed in Table 2. The Cr(VI) removal (%) was in the range of 0–93.3%—corresponding to experimental runs 1 and 22, respectively (Table 2). These two experiments were performed at the same temperature (30 °C) and initial Cr(VI) concentration (50.5 mg/L) but used different adsorbent dosages (0 and 2 t-TW g L^−1^) and pH values (7 and 2). From the experimental results, an empirical model representing the relationship between the operating factors and Cr(VI) removal (%) response was developed:

2 $$ {\text{Cr}}\left( {{\text{VI}}} \right){\text{ removal }}\left( \% \right) \, = { 9}.{56 } - { 22}.{\text{13 A }} - { 15}.0{\text{2 B }} + { 4}.{\text{57 C }} + { 1}.{\text{86 D }} + { 2}.{\text{95 AB }} - { 3}.{\text{31 AC }} - { 2}.{\text{26 AD }} - { 3}.{\text{47 BC }} + { 9}.{\text{16 A}}^{{2}} + { 5}.{9}0{\text{ B}}^{{2}} + { 11}.{\text{29 A}}^{{2}} {\text{B }} + { 5}.{4}0{\text{ A}}^{{2}} {\text{C }} + { 3}.{\text{41 A}}^{{2}} {\text{D }} + { 12}.{4}0{\text{ AB}}^{{2}} . $$

**Figure 3:**
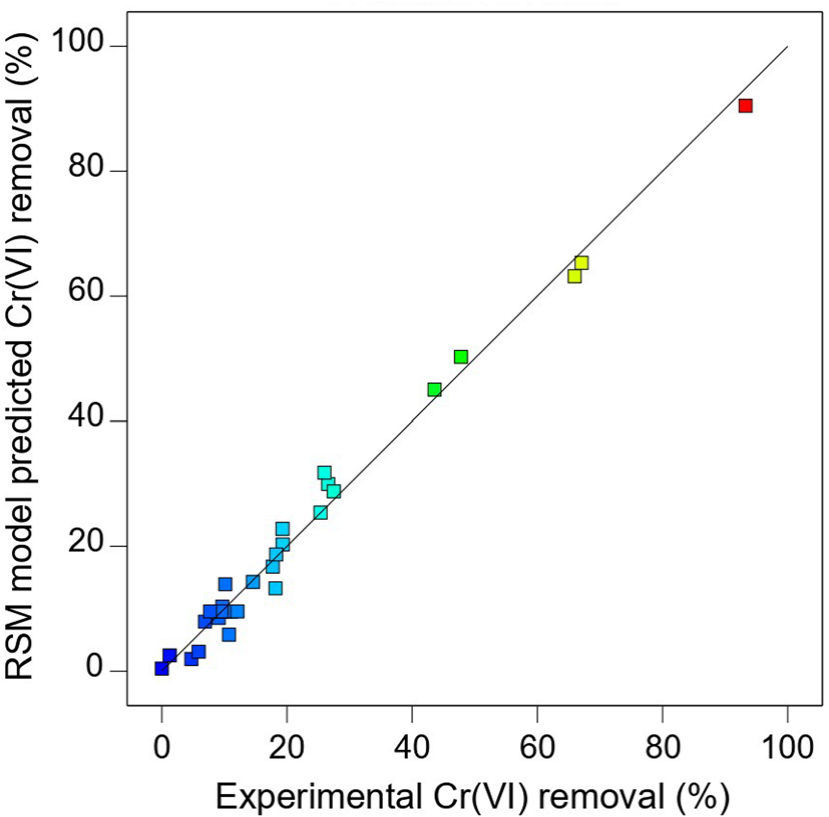
Predicted versus experimental plot for Cr(VI) removal by ascorbic acid treated tea waste (t-TW). Experimental conditions are provided in Table 2.

The coefficients in the equation represent the linear, quadratic, and cubic terms of the factors. A negative sign indicates an antagonistic effect, whereas a positive term denotes a synergistic effect of a certain factor (or combination of factors) on Cr(VI) removal (%)^21,33^. The adequacy of the model (Eq. (2)) to represent the experimental data was tested by plotting the experimental values against values predicted by the RSM model. The RSM model exhibited satisfactory approximation of the actual Cr(VI) adsorption removal (%), as demonstrated by the high correlation coefficient (R^2^) of 0.99 (Fig. 3), implying that 99% of the variations in the results can be attributed to the studied factors^34,35^. In addition, the adjusted correlation coefficient (adj. R^2^ = 0.97) was close to the R^2^. An adj. R^2^ is usually preferred over R^2^ because, unlike R^2^, an adjusted value only increases upon the addition of statistically significant model term(s)^21^. The difference between the adj. R^2^ and predicted R^2^ was 0.23, which was slightly higher than the desirable range (≤ 0.2)^36^. This may be due to the complexity of the cubic model and suggests that the model should be used with caution when predicting above or below experimentally validated ranges.

Analysis of variance (ANOVA) was performed to statistically validate the model, as shown in Table 3. The model and all its terms (except “D”) were statistically significant at a > 95% confidence level, indicated by p values below 0.05. The linear term D, representing the effect of temperature on Cr(VI) reduction, was significant only at an 85% confidence level. Despite its statistically low significance, it was retained to maintain the model’s hierarchy due to its interaction with the statistically significant terms (AD and A^2^D)^37^. The adequate precision (signal-to-noise ratio) was 36.56, which is well above the required threshold value of 4. This indicates adequate response signals and the suitability of the model for use in the experimental design space^21,38^. The regression analysis and ANOVA therefore validate the model as an appropriate tool to study the effect of factors on Cr(VI) adsorption removal.

**Table 3:** Analysis of variance of a reduced cubic model representing adsorptive removal of ascorbic acid treated tea waste.

Source of variation	Sum of squares	Degrees of freedom	Mean square	F value	p value	Significance
Model	13,354	14	954	79	< 0.0001	Significant
A:pH	3919	1	3919	323	< 0.0001	
B:Cr-6	1804	1	1804	149	< 0.0001	
C:t-TW	167	1	167	14	0.0021	
D:Temp.	28	1	28	2	0.1523	
AB	139	1	139	11	0.0041	
AC	176	1	176	14	0.0017	
AD	82	1	82	7	0.0202	
BC	193	1	193	16	0.0012	
A^2^	2389	1	2389	197	< 0.0001	
B^2^	989	1	989	82	< 0.0001	
A^2^B	680	1	680	56	< 0.0001	
A^2^C	156	1	156	13	0.0027	
A^2^D	62	1	62	5	0.0393	
AB^2^	820	1	820	68	< 0.0001	
Residual	182	15	12			
R^2^	0.99		Adj. R^2^	0.97		
Predicted R^2^	0.74		Adeq. precision	36.56		

### Effects of variables on Cr(VI) adsorption

**Figure 4:**
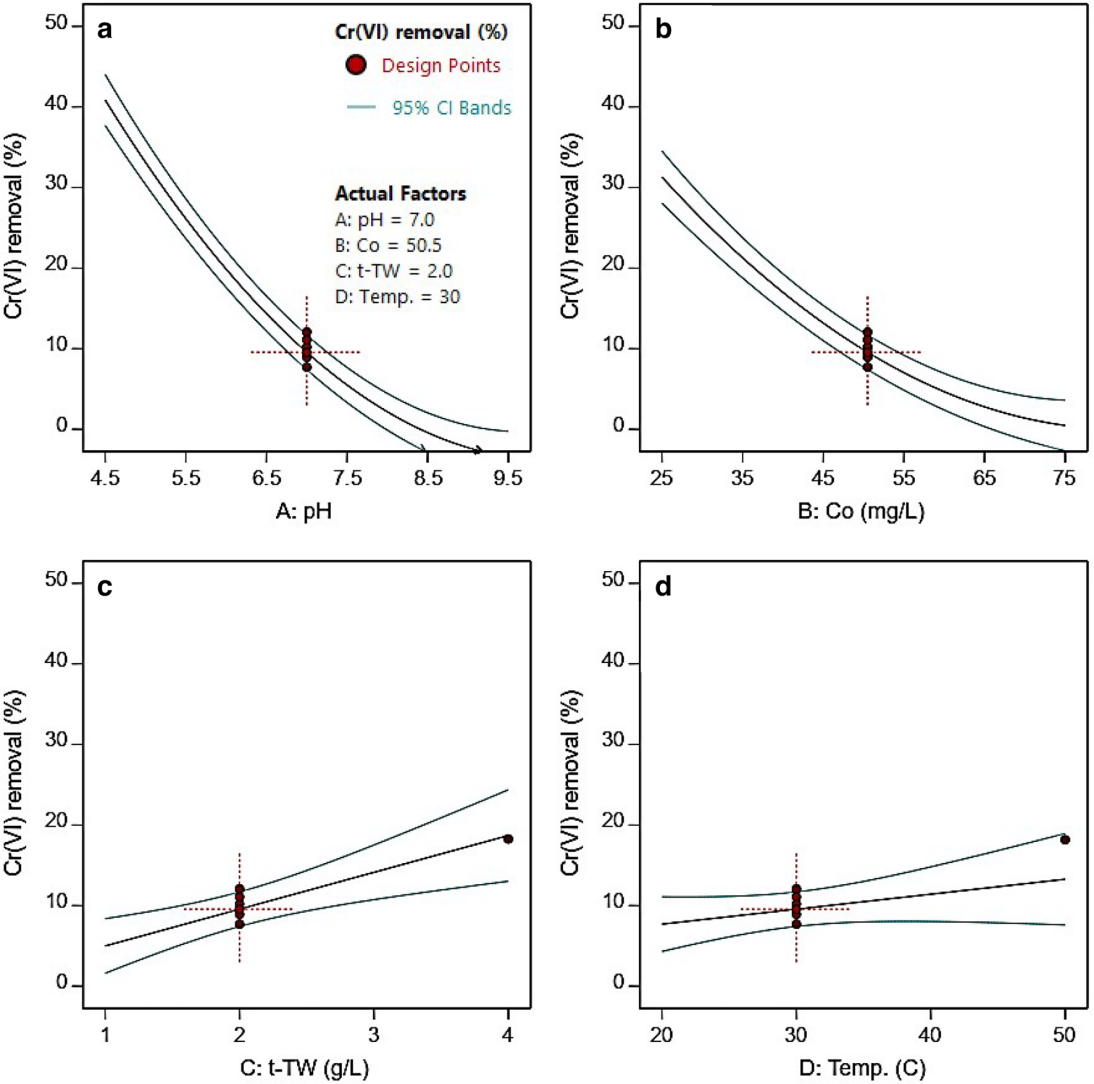
One-factor effects plots of (**a**) pH, (**b**) initial Cr(VI) concentration, (**c**) t-TW dosage, and (**d**) temperature on Cr(VI) adsorption from water.

To investigate the linear effects of changing the levels of a single factor on the response, one-factor effects plots were generated (Fig. 4). Cr(VI) removal was effected by pH, initial Cr(VI) concentration, adsorbent (t-TW) dosage, or temperature. The red circles represent experimental design points, black lines represent modeled prediction, and turquoise lines represent the least-significant-difference at a 95% confidence level. Figure 4a shows that Cr(VI) removal decreased with an increase in pH from 4.5 to 9.5 (level ± 1 from Table 1). At a pH of 4.5, approximately 40% Cr(VI) removal was observed, and this decreased to 10 ± 2% as the pH approached neutrality. The trend continued at basic pH conditions until no removal was observed at pH 8.5. The decrease in Cr(VI) adsorption with an increase in pH has been reported in activated carbons^39^, nanocomposites^40^, and organic adsorbents^41^ such as ours. The pH of the solution affects the speciation of metal ions as well as the surface charge of the adsorbent. The point of zero charge of TW is close to a pH of 5.7^42^. The Cr(VI) speciation in aqueous solution is driven by pH^40^. At pH ≤ 1, Cr(VI) exists primarily as chromic acid (H_2_CrO_4_). At pH 1–7, the hydrogen chromate (HCrO_4_^–1^) ion dominates, whereas above pH 7 only the chromate ion (CrO_4_^–2^) prevails^40,43,44^. The gradual decrease of adsorption from acidic to neutral pH can be explained by the gradual conversion of predominantly monovalent hydrogen chromate (HCrO_4_^–1^) to divalent chromate (CrO_4_^–2^, Cr_2_O_7_^–2^) ions. Because the free energy of adsorption of divalent ions is higher than that of monovalent ions, the divalent ions adsorbed less frequently on t-TW^40^. The surface of the t-TW was also deprotonated with an increase in the pH of the surrounding aqueous solution, leading to decreased positive surface charges. Consequently, the negatively charged chromate ions experienced electrostatic repulsion at a higher pH, which resulted in decreased adsorption of chromate ions.

Figure 4b depicts the decline in Cr(VI) removal (%) as the initial Cr(VI) concentration increases from 25.5 to 50.5 (mg L^−1^). The trend is similar to that of pH, but less pronounced (a less steep slope) when compared with pH. The decrease in adsorption removal—upon increasing initial Cr(VI) concentrations—can be attributed to the unavailability of sufficient adsorption sites (on t-TW) at higher initial Cr(VI) concentrations^43^. This hypothesis was supported by the increase in Cr(VI) removal with an increase in t-TW dosage, as shown in Fig. 4c, with a nearly linear increase in Cr(VI) adsorption occurring as the t-TW dosage increased. Figure 4d shows an insignificant increase in Cr(VI) removal with an increase in temperature from 20 to 50 °C. A small (0.7%) increase in Cr(VI) removal was observed upon increasing the temperature from 20 to 30 °C, which is difficult to attribute to an increase in mass transfer rate with an increase of temperature^45^. In addition, the term is significant only at an 85% confidence level (Table 3), and such a minor change can be considered inconsequential. To summarize, one-factor-at-a-time plots suggest (i) the greatest impact on Cr(VI) removal was pH followed by initial Cr(VI) concentration, adsorbent dosage, and temperature, (ii) an increase in Cr(VI) removal from water with a decrease in pH and initial Cr(VI) concentration, and (iii) an increase in Cr(VI) removal from water with an increase in the adsorbent dosage.

**Figure 5:**
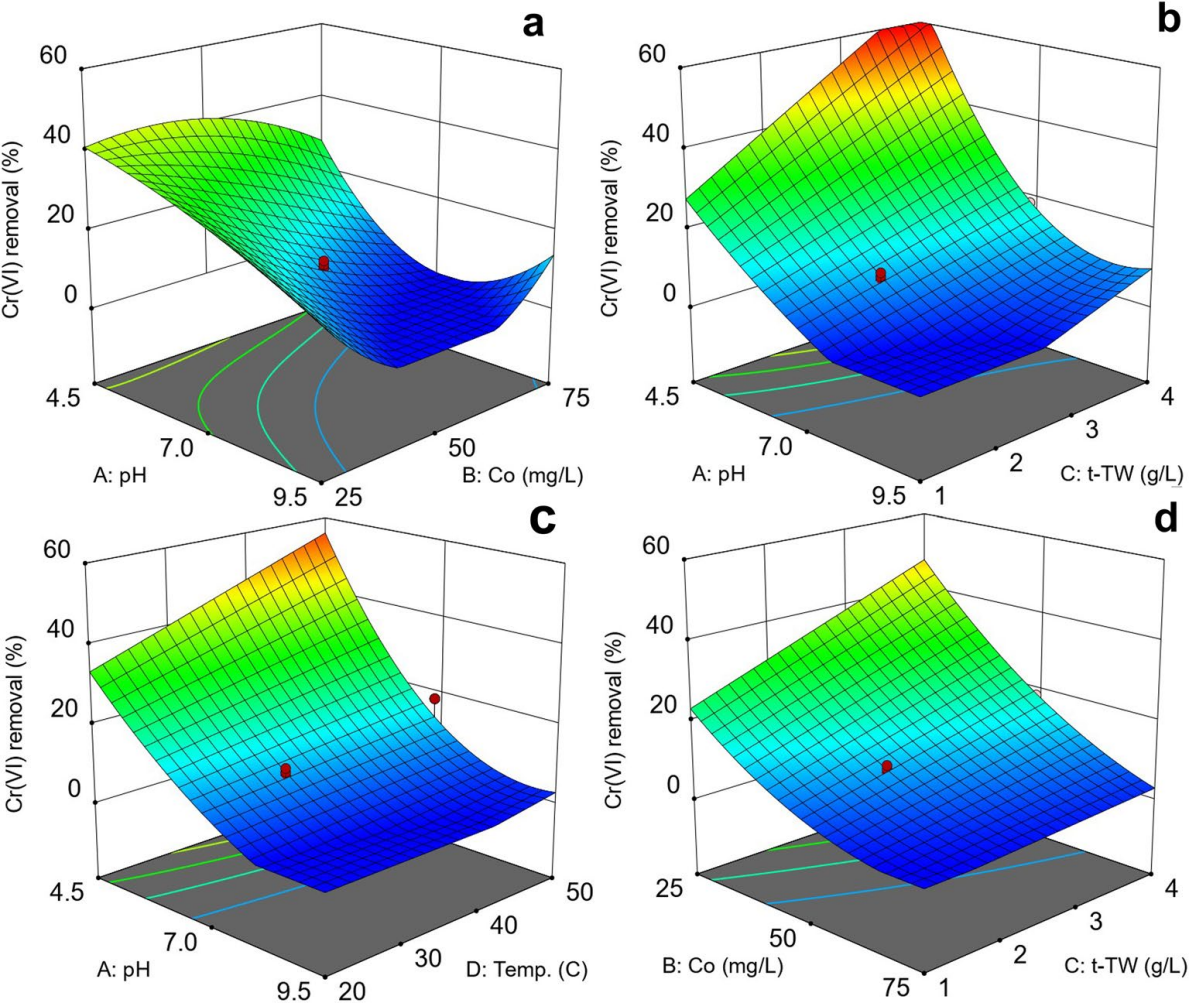
Combined effects of factors on Cr(VI) adsorption removal from water: (**a**) pH and initial Cr(VI) concentration at a t-TW dosage of 2 g L^−1^ and a temperature of 30 °C, (**b**) pH and t-TW dosage at an initial Cr(VI) concentration of 50.5 mg L^−1^ and a temperature of 30 °C, (**c**) pH and temperature at an initial Cr(VI) concentration of 50.5 mg L^−1^ and a t-TW dosage of 2 g L^−1^, and (**d**) initial Cr(VI) concentration and t-TW dosage at a pH 7 and a t-TW dosage 2 g L^−1^.

Further analysis of the model parameters was performed using three-dimensional response surface plots. An RSM allows for the investigation of the combined effects of factors on response with the aid of surface plots. Surface plots can be generated by varying two variables at a time and observing their effect on the response, while keeping the others constant at a certain level (usually mid-range)^33,46^. Table 3 lists the significant interactions of the terms AB, AC, AD, and BD, and response surfaces were generated to study these interactions. Figure 5 shows the combined effects of pH and initial chromium concentration (AB), pH and adsorbent dosage (AC), pH and temperature (AD), and initial chromium concentration and adsorbent dosage (BC). A decrease in Cr(VI) adsorption removal can be seen with an increase in pH in combination with the initial Cr(VI) concentration (Fig. 5a), t-TW (Fig. 5b), and temperature (Fig. 5c). The pH-dominated effects, such as the one-factor effect of pH (Fig. 4a), can be explained by the prevalence of fewer affinitive chromate ions (at high pH), leading to less-frequent electrostatic interactions with the adsorbent surface, resulting in reduced adsorptive removal of Cr(VI)^39,40^. Figure 5d shows the decrease in adsorptive removal with a decrease in adsorbent dosage and increase in initial Cr(VI) concentrations. This may be due to the unavailability of adsorption sites for Cr(VI) adsorption at lower t-TW dosages, as discussed earlier^39^. To conclude, one-factor plots and response surface graphs establish the most pronounced effect of pH on Cr(VI) removal, which is consistent with earlier similar studies^43,47^. These observations require the optimization of parameters to effectively remove Cr(VI) from water using t-TW in a pH range suitable for drinking water.

### Process optimization

**Figure 6:**
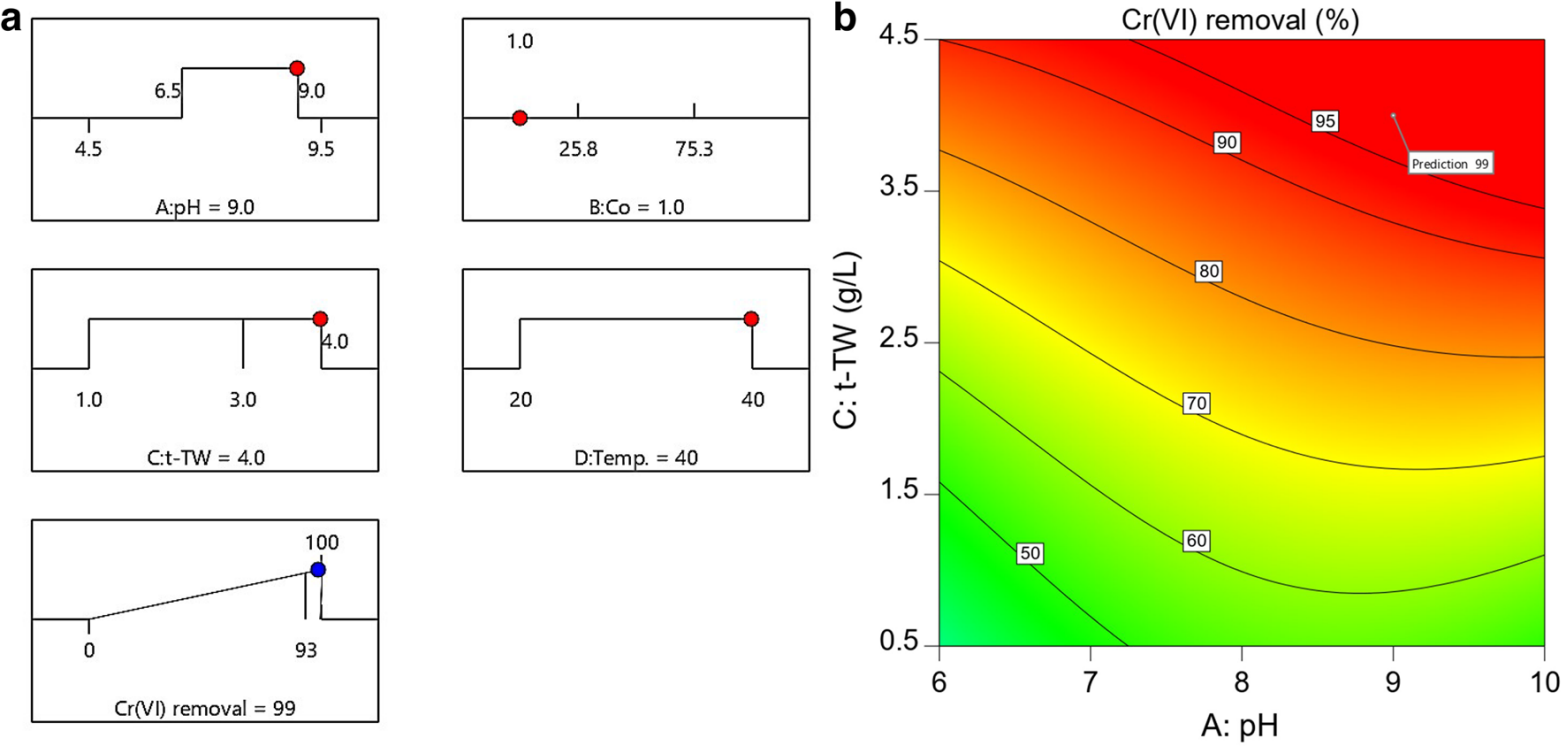
(**a**) Ramp plots and (**b**) a contour plot representing optimized conditions for ≥ 99% removal of Cr(VI) from water. The flag indicates the location of parameters for optimized removal. (Contact time: 48 h; initial pH: 9.0; initial Cr(VI) concentration; 1.0 mg L^−1^; adsorbent dosage: 4.0 g L^−1^; and temperature: 40 °C).

The adsorption removal of Cr(VI) was optimized in drinking water pH range (6.5–9.5) using t-TW as an adsorbent. The initial Cr(VI) concentration was fixed at 1 mg L^−1^ and complete removal was targeted as shown in ramp plots (Fig. 6a). The model predicted 99% removal, thereby limiting the residual concentration of Cr(VI) to 0.01 mg L^−1^. The targeted residual Cr(VI) concentration—at optimized conditions—was well below the allowable concentrations recommended by the US EPA (0.1 mg L^−1^) and WHO (0.05 mg L^−1^)^5,6^. Experiments were conducted at prescribed settings and no residual Cr(VI) was detected. A ≥ 99% adsorption removal of Cr(VI) at optimized conditions was assumed due to the method detection limit of 0.002 mg L^−1^. The flag point in Fig. 6b represents the location of optimized parameters in the experimental design space.

### Adsorption equilibrium

The established isotherm models were fitted to the experimental data to determine the adsorption of Cr(VI) by t-TW. The mathematical expressions describing Langmuir, Freundlich, Temkin, and D-R models are:

3 $$Langmuir: {q}_{e}=\frac{{q}_{m}{K}_{L}{C}_{e}}{1+ {K}_{L}{C}_{e}},$$

4 $$Freundlich: {q}_{e}={K}_{f}{{C}_{e}}^\frac{1}{n},$$

5 $${Temkin: q}_{e}=\frac{RT}{{b}_{T}}\text{ln}\left({A}_{T}{C}_{e}\right),$$

6 $$Dubinin{-}Radushkevich :{q}_{e}={q}_{DR}{e}^{-{K}_{DR}{\varepsilon }^{2}},$$

where ${C}_{e}$ (mg L^−1^) is the equilibrium concentration, ${q}_{m}$ (mg g^−1^) is the Langmuir maximum adsorption capacity, ${K}_{L}$ (L mg^−1^) is the Langmuir adsorption constant, ${K}_{f}$ ([mg g^−1^] [L mg^−1^] ^1/n^) is the Freundlich constant, n is the Freundlich exponent, ${A}_{T}$ (L.mg^−1^) is the Temkin isotherm equilibrium binding constant, ${b}_{T}$ (J mol^−1^) is the Temkin isotherm constant, ${q}_{DR}$ (mg g^−1^) is the D-R maximum sorption capacity, ${K}_{DR}$ (mol^2^ kJ^−2^) is the D-R constant related to sorption energy, and $\varepsilon$ (= RT ln [1/1 + ${C}_{e}]$) is the Polanyi potential.

**Figure 7:**
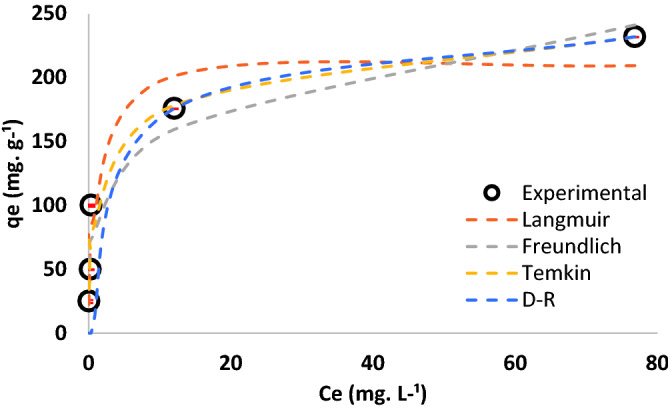
Comparison of equilibrium experimental data with the Langmuir, Freundlich, Temkin, and Dubinin–Radushkevich (D–R) models. (Contact time: 48 h; initial pH: 6.5; initial Cr(VI) concentration: 100 mg L^−1^; adsorbent dosage: 0.1–4 g L^−1^; and temperature: 25 °C).

Figure 7 shows Cr(VI) adsorption isotherms and the corresponding equilibrium parameters reported in Table 4. The adsorption equilibrium was best described by Temkin > Langmuir > Freundlich > D-R models, based on regression coefficients. The Temkin model assumes uniform distribution of binding energy sites on t-TW surface and a linear decrease in the heat of adsorption of Cr(VI) species as the adsorption progressed^23,48^. Similarly, the Langmuir model also suggests monolayer adsorption^23,49^. The calculated separation factor suggests Langmuir adsorption of Cr(VI) on t-TW was favorable^48^. Nevertheless, Freundlich and D-R fittings were also significant which indicates multilayer adsorption and pore filling due to heterogeneous surfaces^23,48^. Therefore, it is assumed that the adsorption of Cr(VI) on t-TW was largely monolayer, but occasionally multilayer, in nature due to a mixture of uniform and non-uniform surfaces, as shown in SEM micrographs (Fig. 1).

**Table 4:** Adsorption equilibrium and kinetics parameters for Cr(VI) adsorption onto tea waste treated with ascorbic acid.

Model	Parameters	R^2^
Langmuir	K_L_ = 1.75 (L mg^−1^); q_m_ = 210.97 (mg g^−1^);	0.95
Freundlich	K_F_ = 91.39 (mg g^−1^). (L g^−1^)^4.5^; n = 4.50	0.94
Temkin	b_T_ = 79.94 (kJ mol^−1^); A_T_ = 39.90 (L g^−1^)	0.97
Dubinin–Radushkevich (D–R)	E_DR_ = 266.00 (J mol^−1^); q_DR_ = 233.70 (mg g^−1^); K_DR_ = 7.05 (mol^2^ k^−1^ J^−2^)	0.90
PFO	k_1_ = 0.12 (1 h^−1^); q_e_ = 159.00 (mg g^−1^)	0.95
PSO	k_2_ = 0.001 (g mg^−1^ h^−1^); q_e_ = 176.00 mg g^−1^	0.98
Intraparticle	K_IP(1)_ = 34.52 (mg g h^−1^), C_(1)_ = 0.81 (mg g^−1^), K_IP(2)_ = 0.68 (mg g h^−1^), C_(2)_ = 111.00 (mg g^−1^)	0.97, 0.96

### Adsorption kinetics

**Figure 8:**
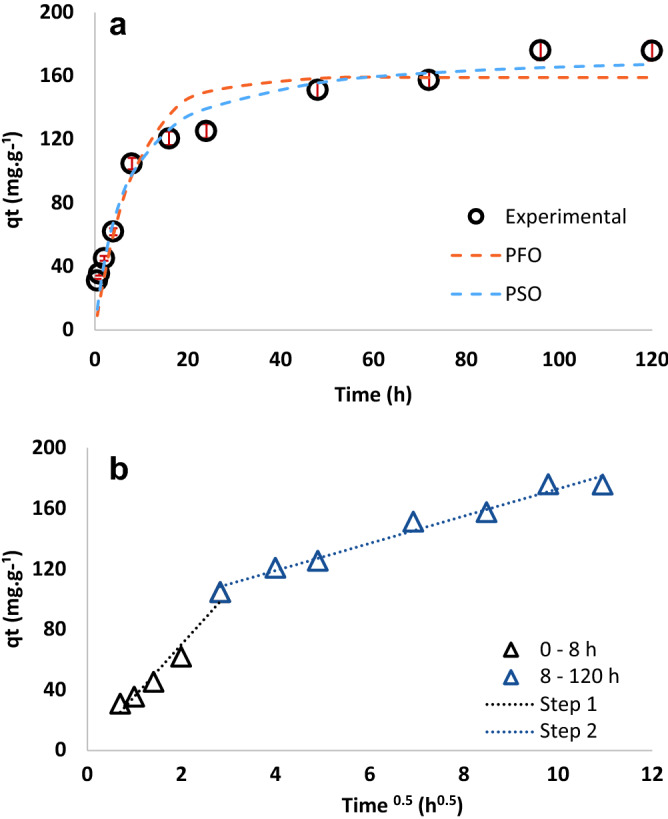
Comparison of experimental kinetic data with (**a**) pseudo-first-order (PFO) and pseudo-second-order (PSO) kinetics models, and (**b**) an intraparticle diffusion model. (Contact time: 0.5–120 h, initial pH: 6.5; initial Cr(VI) concentration: 100 mg L^−1^; adsorbent dosage: 0.5 g L^−1^; and temperature: 25 °C).

Adsorption kinetics describe the rate and mechanism of the adsorption process. Figure 8 shows the saturation of t-TW surface with Cr(VI) over time. Adsorption kinetics were rapid for the first 8 h, likely due to the abundance of available adsorption sites for the initial adsorption. Afterward, adsorption of Cr(VI) progressed at a relatively slower rate from 8 to 24 h and reached equilibrium after 2 days. The experimental kinetic data were fitted to the PFO, PSO, and intraparticle models^24^:

7 $${\text{PFO}: q}_{t}={q}_{e}\left(1-{e}^{-{k}_{1}t}\right),$$

8 $${\text{PSO}: q}_{t}=\frac{t{k}_{2}{q}_{e}^{2}}{1+{q}_{e}t{k}_{2}},$$

9 $${\text{Intraparticle diffusion}: q}_{t}={K}_{ip}{t}^{0.5}+C,$$

where ${q}_{t}$ is the adsorption capacity at time t, ${k}_{1}$ (1 h^−1^) is the PFO rate constant, ${k}_{2}$ (g mg^−1^ h^−1^) is the PSO rate constant, ${K}_{ip}$ (mg g^−1^ h^−0.5^) is the intraparticle diffusion rate constant, and C (mg g^−1^) is the intercept of intraparticle diffusion plot.

Figure 8 shows PFO, PSO, and intraparticle diffusion kinetic models fitted to experimental data. Their corresponding kinetic parameters are tabulated in Table 4. The PSO kinetic model appears to fit the data reasonably well as evident from a high regression coefficient value of 0.98^25^. Because the PSO model closely predicted the experimental equilibrium adsorption capacity, it was assumed that Cr(VI) adsorption by t-TW was a physisorption phenomenon in which the adsorption rate was proportional to the availability of adsorption sites^24,25^.

The intraparticle diffusion model employs an empirical relationship that relates the amount of adsorbate to the square root of time (t^0.5^), as shown in Eq. (9)^25^. The adsorption of Cr(VI) by t-TW generated two-step multi-linear intraparticle diffusion plots (Fig. 8b): step 1 from 0 to 8 h and step 2 from 8 to 120 h. Nearly 60% of Cr(VI) was adsorbed by the t-TW during step 1. Also, the slope of step 1 is steeper than that of step 2, indicating rapid transport of Cr(VI) ions from the bulk to the external surface of t-TW^24,25^. Corresponding parameters of the intraparticle diffusion plots are provided in Table 4. The intraparticle model fits the experimental data reasonably well as evident by high regression coefficients. The intraparticle diffusion model postulates that intraparticle diffusion would be the sole rate-limiting step if the plot of qt versus t^0.5^ crosses the origin^24^. The intercept of the second step did not pass from the origin, suggesting that several mechanisms are involved and intraparticle diffusion is not the sole rate-limiting step^25^.

### Comparison with other adsorbents

The adsorption capacity of t-TW compared with other adsorbent materials reported in the literature is presented in Table 5. The adsorption capacity of t-TW is 232 mg g^−1^, which is significantly higher than several low-cost and some advanced adsorbents. Moreover, ascorbic acid treatment to synthesize t-TW is a simple process that requires no pre-treatment of the adsorbent surface. The pre-treatment step is often an energy-intensive process that requires the use of hazardous and corrosive chemicals, such as persulfates, mineral acids, peroxides, and alkalis^14,50^. Ascorbic acid–mediated t-TW synthesis can therefore be classified as a green route toward material functionalization.

**Table 5:** Comparison of adsorption of Cr(VI) with other adsorbents.

Sr. no	Adsorbent	Cr (VI) (mg g^−1^)	Ref.
1	Rice husk	52.1	Sugashini et al.^51^
2	Tamarind hull	81.1	Verma et al.^52^
3	Industrial waste	15.2	Gupta et al.^53^
4	Ionic solid impregnated phosphate chitosan	266.7	Kahu et al.^54^
5	KOH-activated activated carbon	315.0	Khezami and Capart^50^
6	Chitosan/aluminum–lanthanum mixed oxyhydroxide (CSALMOH)	78.9	Preethi et al.^55^
7	Mesoporous silica embedded with magnetite nanoparticles	50.5	Hozhabr Araghi et al.^56^
8	Red algae	26.5	Sari and Tuzen^8^
9	Tea waste biochar	198.0	Khalil et al.^14^
10	Ascorbic acid treated tea waste	232.2	This study

## Conclusions

The utilization of tea waste (TW) as an adsorbent material can help reduce solid waste disposal problems, thereby contributing to sustainable consumption and production objective. In this study, treated tea waste (t-TW) was prepared from spent black TW by modifying it with ascorbic acid; an environmentally friendly, non-toxic, and inexpensive functionalizing agent. The experimental adsorption capacity of t-TW was close to 232 mg g^−1^, one of the highest among agricultural waste-based adsorbents. The synthesized t-TW was investigated for toxic Cr(VI) removal from aqueous systems using a statistical experimental design and an RSM. The empirical model was developed and statistically validated to describe the effect of pH (2–12), initial Cr(VI) concentration (1–100 mg L^−1^), adsorbent dosage (0–4 g L^−1^), and temperature (10–50 °C) on adsorption removal of Cr(VI) from aqueous systems. The adsorption removal of Cr(VI) was chiefly regulated by the pH of the solution. The RSM model was used to predict (and later experimentally verify) ≥ 99% removal of Cr(VI) from water at optimized conditions; pH = 9; initial Cr(VI) concentration = 1 mg L^−1^; adsorbent dosage = 4 g L^−1^; and temperature = 40 °C. The residual Cr(VI) concentrations after treatment, at optimized conditions, comply with US EPA and WHO regulatory requirements. The adsorption equilibrium data were best fitted to Temkin and Langmuir isotherms, indicating monolayer adsorption on heterogeneous surface. The kinetics were adequately described by PSO and intraparticle diffusion models. The adsorption of Cr(VI) was controlled by intraparticle diffusion on the t-TW surface. The results indicate that t-TW is a promising candidate for the adsorptive removal of heavy metals from aqueous systems.

## Data Availability

The datasets used and/or analyzed during the current study available from the corresponding author on reasonable request.
